# Radiological Scouting, Monitoring and Inspection Using Drones

**DOI:** 10.3390/s21093143

**Published:** 2021-04-30

**Authors:** Luís Ramos Pinto, Alberto Vale, Yoeri Brouwer, Jorge Borbinha, José Corisco, Rodrigo Ventura, Ana Margarida Silva, André Mourato, Gonçalo Marques, Yuri Romanets, Susana Sargento, Bruno Gonçalves

**Affiliations:** 1 Insituto de Plasmas e Fusão Nuclear, Instituto Superior Técnico, Universidade de Lisboa, Av. Rovisco Pais 1, 1049-001 Lisboa, Portugal; avale@ipfn.tecnico.ulisboa.pt (A.V.); ybrouwer@ipfn.tecnico.ulisboa.pt (Y.B.); bruno@ipfn.tecnico.ulisboa.pt (B.G.); 2 Centro de Ciências e Tecnologias Nucleares, Instituto Superior Técnico, Universidade de Lisboa, Estrada Nacional 10, ao km 139,7 2695-066 Bobadela, Portugal; jorgeborbinha@ctn.tecnico.ulisboa.pt (J.B.); corisco@ctn.tecnico.ulisboa.pt (J.C.); yuriy@ctn.tecnico.ulisboa.pt (Y.R.); 3 Institute for Systems and Robotics, Instituto Superior Técnico, Universidade de Lisboa, Av. Rovisco Pais 1, 1049-001 Lisboa, Portugal; rodrigo.ventura@isr.tecnico.ulisboa.pt; 4 Instituto de Telecomunicações, University of Aveiro, Campus Universitário de Santiago, 3810-193 Aveiro, Portugal; margaridaocs@ua.pt (A.M.S.); andremourato@ua.pt (A.M.); gjmarques@ua.pt (G.M.); susana@ua.pt (S.S.)

**Keywords:** 3D reconstruction, drone, GMC, CZT, heatmap, radiological inspection, radiological sensor, SLAM, UAV

## Abstract

Human populations and natural ecosystems are bound to be exposed to ionizing radiation from the deposition of artificial radionuclides resulting from nuclear accidents, nuclear devices or radiological dispersive devices (“dirty bombs”). On the other hand, Naturally Occurring Radioactive Material industries such as phosphate production or uranium mining, contribute to the on site storage of residuals with enhanced concentrations of natural radionuclides. Therefore, in the context of the European agreements concerning nuclear energy, namely the European Atomic Energy Community Treaty, monitoring is an essential feature of the environmental radiological surveillance. In this work, we obtain 3D maps from outdoor scenarios, and complete such maps with measured radiation levels and with its radionuclide signature. In such scenarios, we face challenges such as unknown and rough terrain, limited number of sampled locations and the need for different sensors and therefore different tasks. We propose a radiological solution for scouting, monitoring and inspecting an area of interest, using a fleet of drones and a controlling ground station. First, we scout an area with a Light Detection and Ranging sensor onboard a drone to accurately 3D-map the area. Then, we monitor that area with a Geiger–Müller Counter at a low-vertical distance from the ground to produce a radiological (heat)map that is overlaid on the 3D map of the scenario. Next, we identify the hotspots of radiation, and inspect them in detail using a drone by landing on them, to reveal its radionuclide signature using a Cadmium–Zinc–Telluride detector. We present the algorithms used to implement such tasks both at the ground station and on the drones. The three mission phases were validated using actual experiments in three different outdoor scenarios. We conclude that drones can not only perform the mission efficiently, but in general they are faster and as reliable as personnel on the ground.

## 1. Introduction

Chemical, Biological, Radiological and Nuclear (CBRN) threats are increasingly present due to wars, terrorist attacks, disasters or simply due to negligence and non-compliance of some human activities. In a work developed by the National Academy of Engineering (USA) [1], the prevention of nuclear terror was identified as one of the major challenges for the 21st century. Radiological threats may impact populations on a daily basis.

First, there are Naturally Occurring Radioactive Materials (NORM) scenarios resulting from residual deposits of phosphate production and uranium mining, with enhanced concentrations of uranium decay products [2]. Due to erosion, rain and other natural weather phenomena, those natural radionuclides contained in landfills may be remobilized in the ecosystems and reach human populations directly or indirectly via their agricultural and animal economies. Ore mining, such as Uranium mining, produces a great quantity of tailings, to be stacked on site. It is common to find radiation levels above background on such NORM sites. Secondly, the the possibility of unforeseen events must be envisaged [3], such as: nuclear plant disasters (Chernobyl, 1986 and Fukushima, 2011), illegal or negligent disposal of radioactive material (Goiânia accident, 1987) and nuclear terrorism attacks using radiological dispersal devices (“dirty bombs”). Regarding all the above mentioned circumstances, we have now a varied set of scenarios each of them with its particular magnitude, meaning potential threats due to the spread of radioactive contamination and the consequent exposure of the environment and human populations to ionizing radiation.

The launching of European Atomic Energy Community (EURATOM) Treaty in the European Union [4] set up the present policies of radiological protection and safety, including the commitment that “each Member State shall establish the facilities necessary to carry out continuous monitoring or the level of radioactivity in the air, water and soil and to ensure compliance with the basic standards (Article 35)”. Public environment agencies and mining enterprises prioritize sensing technologies that are easily transported to a target scenario and set up in a short period of time to retrieve the most relevant information in the field during or immediately after sampling. Currently, such locations (e.g., abandoned ore mines) are routinely surveyed and mostly have been remediated. Personnel move on foot, carrying hand-held Geiger–Müller Counters (GMCs) that register random events of nuclei-fission as count rates (counts per minute, CPM), and/or gamma radiation spectrometers (such as a Cadmium–Zinc–Telluride detector (CZT) to identify and quantify the gamma emitters. These sensors allow staff to check for locations with radiation above nominal values. This process is tedious, time-consuming and typically limited to places humans can access. Furthermore, agencies have too many locations under their jurisdiction where it is their responsibility to carry out radiological surveys, and little human resources to do it as often and thoroughly as desired.

Topographic maps or other representations of the scenario are typically not available (especially in digital formats), or are outdated mainly given unpredictable situations (e.g., scenario changes due to natural causes or human intervention). Nevertheless, such agencies view in great regard the possibility of operators interacting with 3D maps that overlay Heatmaps—maps that code radiation level by color, and Hotspots—locations of highest radiation and their radionuclide signature.

A map is also important for evaluating the topographical changes over time in a specific area, where regular inspections are performed. Lastly, assuming 3D maps are detailed and precise, they can become a valuable resource and allow other drones or agents in general to navigate in the scenario with simpler and cheaper sensor systems in posterior missions.

In this work, we target outdoor scenarios of operation that are not mapped nor characterized, or may have suffered recent topography changes due to natural or human causes, including disasters. We propose to use a fleet of Unmanned Aerial Vehicle (UAVs) in a cooperative fashion to perform such activities. A rotary-wing UAV, commonly known as a multirotor, or in this paper referred to as a drone, is the most suitable solution mainly due to the ability to hover, therefore providing closer proximity to sources and long measurement time on the same location. Under the Fleet of Drones for Radiological Inspection, Communication and Rescue (FRIENDS) project, we aim to develop a new set of algorithms for drones to navigate autonomously and collect radiological data from the scenario, and algorithms to process collected data to generate radiological information such as heatmaps [5]. Fast and powerful processing algorithms can provide the ability to get results and assess conclusions in loco and, if necessary, perform additional manual inspection operations in the field.

Unlike personnel, drones can enter complex terrain by air, fly over obstacles, carry heavy sensors and measure data for long periods of time without physical exhaustion and repeat such tasks without mental exhaustion, either. Last but not least, drones are expendable under the presence of higher than human-safe radiation. Drones are able to carry out different missions of radiological inspection in most of the scenarios with minimal disturbance to the scenario.

However, the major challenge in using drones for such operations is to guarantee a reliable performance in detecting radioactivity, at least as well as human operator carrying a radiological probe. Drones are limited in the time of flight, they need regular maintenance, they cannot access the floor-level under bushes or other thick vegetation, and they necessarily must carry small radiological sensors due to their payload constraints.

In this paper, we claim that drones can be used to quickly generate complete and reliable radiological 3D maps in outdoor scenarios. We envision such maps being complemented by others made by traditional methods. The authors’ proposal is a solution able to jointly:

- Generate a 3D map of scenario using Drones
- Collect radiological data captured by Drones
- Automatically identify hotspots and characterize its radionuclides’ signature.

In this paper, we propose new guidelines for a three-stage radiological UAV survey mission, and new methods to generate 3D maps of radiation in wide outdoor areas using a sparse and finite set of radiological measurements using only drones for data collection, that is sensitive to height from the ground. To the best of our knowledge it has not been done before.

The remainder of the paper is organized as follows. In Section 2, we present some of the related work that is found in the literature regarding radiological monitoring and UAVs. Section 3 starts describing the problem of radiological exploration of scenarios and follows with a proposal for a solution divided into three phases. Section 4 describes the hardware and software architectures used. Section 5 explains the newly-developed algorithms. The experimental results are presented in Section 7, where the hardware employed and the testing scenarios are firstly introduced in Section 6. Finally, the main conclusions and future work are described in Section 8.

## 2. Background and Related Work

Not only is the European Union (EU) aware of the needs for defense against CBRN threats, but also the North Atlantic Treaty Organization (NATO) is aware of the risks of the proliferation of weapons of mass destruction. Rapid advances in biological and technological science continue to increase the threat of bioterrorism against populations. For that, NATO already has a Task Force within the scope of the CBRN, in addition to having lines of financing [6]. An example is the ENCIRCLE project [7], funded by the European Union (EU), which aims to help strengthen the European CBRNe industry (CBRN and Explosive materials, pronounced in English as “C-BURN”). Today, this industry is still somewhat limited and fragmented and, as a result, is not as competitive as it could be in the global playing field [8].

Some authors [9] already proposed some mission guidelines on the use of robots to assist on CBRN incidents. In indoor scenarios, researchers are presenting land robots in general (UGVs) equipped with manipulators to navigate, search and detect radiation and finally collect material if necessary [10]. Multiple robotic trials/competitions have taken place to show new robots and their radiological and nuclear mapping capabilities, such as EnRich [11], ELROB and EURATHLON [12,13] and IAEA Robotic Challenge [14]. In [15], authors provide 3D heatmaps, who use a Unmanned Ground Vehicle (UGV) mounted with a Light Detection and Ranging sensor (LiDAR) and three scintillators for radiation detection. The LiDAR enables terrain mapping and localization, as the authors focus on radiological inspection in Global Navigation Satellite System (GNSS) denied scenarios. Furthermore, two main problems are addressed by the authors: (i) estimating the distributed radiation field given a finite set of measurements and (ii) determining the most informative observation positions and a collision-free path between them. We will use similar solutions, but with a fleet of Unmanned Aerial Vehicle (UAVs).

In outdoor scenarios, several works explored the usage of drones with radiological sensors for such tasks. Drones are widely being used for 3D surveys [16,17]. They typically rely on navigational GNSS data. To improve accuracy and provide autonomous operation, it is common to install RTK systems on board [18,19].

It is more common to find teleoperated systems that collect spatial and radiological data. In [20], authors conclude that UAVs with radiological sensors can be utilized to detect radiation from the ground (nuclear power plant disaster). In the GAMMAex project [21], an aerial system was designed to be used in scenarios where Biological, Chemical and Radiological (BCR) threats are present, through chemical and radiological recognition and monitoring actions. In [22], researchers collect radiological data from wide areas using drones, and there are already commercial solutions, such as [23,24]. In [25], a radiological mission is proposed using drones and ground robots working together to generate 2D heatmaps overlaid on satellite photographs. In neither of these works 3D maps were produced neither 3D heatmaps generated.

In [26], our laboratory has presented preliminary work recreating a scenario in three dimensions using a Red, Green, Blue and Depth (RGBD) camera. A layer of colors was projected on the scenario, according to measured radiation using GMC and CZT sensors. Sensors were all assembled into a portable package. The system was tested using discrete sources and indoors, but it could be used also outdoors and transported by a drone, for example. In [27], authors focus on developing a CZT camera sensors to be on board of drones, and present some their performance, but no flights are presented nor heatmaps generated. Using CZTs onboard UAVs has been tried before [28]. In this work, no georeference is done, nor radionuclide identification.

We will present an architecture integrating some of the concepts from previous works such as using LiDAR to generate 3D maps with Simultaneous Localization and Mapping (SLAM) algorithms, and CZT and GMC for radiological analysis. Furthermore, we will focus on outdoor scenarios and using drones to carry such sensors. A novel aspect of our work is the use of such system for wide-scale radiological measurements of rural landscapes where sources are typically distributed across the ground. This imposes new challenges and new solutions, and for such we will propose new guidelines for a three-phase UAV mission to accomplish such actions. In the end, we propose new methods to generate 3D radiological heatmaps using only data collected by UAVs, that also contain radionuclide information. To the best of our knowledge, it has not been done before.

## 3. Concept of Mission Operation

Given an Area of Interest (AoI), multiple drones with sensing capabilities, and assuming that all radiation is sourced from the ground, our problem is to jointly solve four problems:

1. Produce a 3D map that is able to accurately represent the area segmenting ground and obstacles above the ground.
2. Provide accurate CPM counting of any given location above the ground.
3. Automatically identify the hotspots of radiation on the ground (local maxima).
4. Finally, identify the radionuclides present in the hotspots.

**Figure 1:**
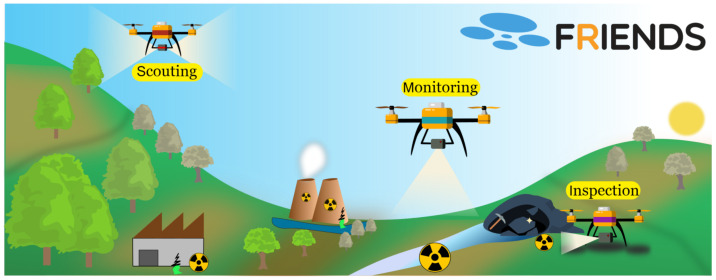
Missions are divided into three major phases: Scouting, Monitoring, Inspection.

To solve this muti-tier problem, we organize our mission in three phases as depicted in Figure 1, namely Scouting to achieve point 1, Monitoring to achieve points 2 and 3, and Inspection to achieve point 4. Each phase encompasses drone flights with different characteristics and goals, followed by an offline data processing procedure.

The first phase—Scouting—is the exploration of the scenario to achieve a 3D representation of it. The success of the overall mission depends first on exploring and characterizing the scenario.

The second phase—Monitoring—regards radiological analysis of the whole scenario based on the map achieved. This phase may run with multiple light-weight drones equipped with radiological sensors (e.g., GMCs) and light-weight obstacle avoidance systems (e.g., RGBD and ultrasound) deployed to thoroughly sweep the selected AoI.

The last phase—Inspection—comprehends a detailed analysis of the radiological activity of the hotspots—the points with highest radiological intensity. These are inspected in detail by landing drones equipped with Gamma spectrometers (e.g., CZT) on such locations.

For now, we assume all phases run sequentially. The Scouting phase is done before the other phases given that the drones in later phases need 3D maps to aid navigation, due to limited onboard sensing capabilities. Limiting onboard sensing, decreases the onboard weight and therefore increases mission time. Monitoring phase may take a long time to perform a thorough sweep of the AoI due to the limited sensitivity (range) of radiological sensors. As the monitoring phase finishes, Inspection phase initiates. The following subsections detail the operation of each one of the three phases.

### 3.1. Scouting Phase

This first phase is designed to perform data acquisition necessary to reproduce a complete 3D point cloud map of the scenario. Using this map, ground and obstacles are segmented. Ground and obstacles will be used in later phases to visualize heatmaps, and also to generate occupancy grids that eventually allow Monitoring drones to navigate using obstacle-free paths.

#### 3.1.1. Flight

We use Scouting drones for this phase. On board is a LiDAR sensor with a spherical view to deliver accurate 3D data. The sensor is placed under the drone as shown in Figure 1 to maximize data received from the ground while flying. An on-board GNSS receiver is also installed to enable us to know the drone location in the AoI and to geo-reference such map.

**Figure 2:**
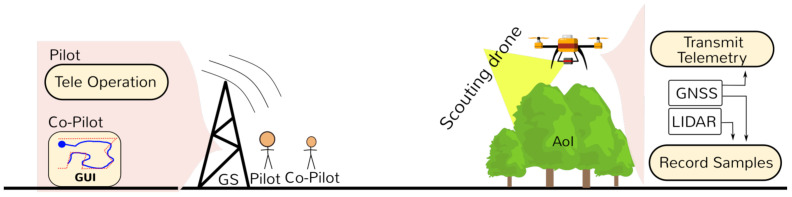
Scouting phase—flight.

The Scouting flight operation is summarized in Figure 2. Once the drone is ready for take-off, the pilot starts the flight, controlling the drone via remote control. During the flight, the co-pilot is able to see the current GNSS position of the drone on a Graphical user Interface (GUI).

Flights cover all the scenario following a boustrophedon path, i.e., sweeping back and forth across the AoI (also known as a lawnmower path). The path has to be compatible to the drone battery range and field of view of the LiDAR.

During flight, LiDAR frames are locally collected along with GNSS and Inertial Measurement Unit (IMU). Due to the typical long range of the LiDAR, and high frequency of acquisition, the flight stage of the scouting mission is a relatively quick process (couple of minutes of flight). It is not necessary to patrol the same area multiple times, nor avoid obstacles, nor fly close to the ground as in the radiological sampling case as it will be explained in Section 3.2.

#### 3.1.2. Post-Flight Processing

**Figure 3:**
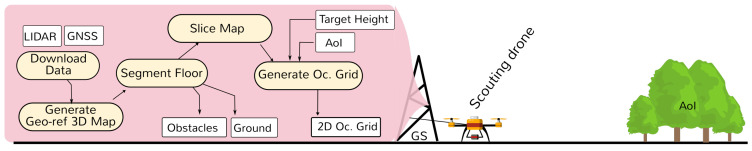
Scouting phase—post-processing: from LIDAR data to 2D Navigation Path.

After the Scouting flight, data are analyzed and processed, and new vital information is produced for the next phase. This process is depicted in Figure 3. The first step initiates when the drone lands. Sensor data recorded during the flight (LiDAR, GNSS and IMU) is downloaded to a computer on the ground—Groundstation (GS).

At the GS, data are processed to retrieve a 3D representation of the scenario, using a SLAM algorithm. If the resulting 3D map is not acceptable to represent the scenario (e.g., areas not covered, important gaps, too many dynamic elements, or low resolution), the previous operation is repeated, where the operator guides the drone along specific areas to mitigate the previous issues. At the end, the new recorded data can be merged (data are georeferenced with GNSS), or completely replaced by the previously acquired data. This operation can be repeated as many times as necessary, usually constrained by the number of spare batteries. At this stage using IMU data, the 3D map is leveled, ie, vertical direction is aligned with the Z-axis. The 3D representation is georeferenced with the GNSS data acquired during the flight, allowing any point in the local referential to be linked to a GNSS coordinate.

The processing phase continues with Segment Floor, i.e., the classification of ground points. All other points are classified as obstacles/objects (such as trees, rocks, etc.). These sets (Obstacles and Ground) are saved for later use. At this stage, we run an 3D free/occupancy algorithm to identify which points can be considered free to navigate, occupied by obstacles, or unknown. Then, the map is sliced. Given the occupancy 3D map and the identified floor-level, this process generates 2D occupancy maps (also known as slices) whose points are at the same height from the ground, within some margin.

Finally, navigational envelopes are generated based on the Target Height and AoI. A slice for the desired height is selected, and cropped based on the chosen AoI. These envelopes represent the set of points where drones can flight obstacle-free at a constant height from the ground. Such data are also known as the 2D Occupancy Grid, and they will be used to perform the next stage of the mission: Monitoring.

### 3.2. Monitoring Phase

The goal of this second phase of the mission is to measure the radioactivity of the AoI. One or more drones can act in cooperation. Moving autonomously, drones sweep the whole AoI at a close distance from the ground. As the radioactivity phenomena decays with squared distance, it is necessary to fly as low and slow as possible. Collected data are georeferenced and placed in the 3D map leading to a 3D heatmap. Furthermore, the list of hotspots to inspect is identified.

#### 3.2.1. Flight

In this part of the mission, we use Monitoring drones (cf. Figure 1). Drones are equipped with a GMC pointing downwards to measure radioactivity, and GNSS to georeference data and allow autonomous flights. These drones are not required to be equipped with LiDAR. They rely on Ultrasound sensors and a RGBD camera to avoid unexpected obstacles.

**Figure 4:**
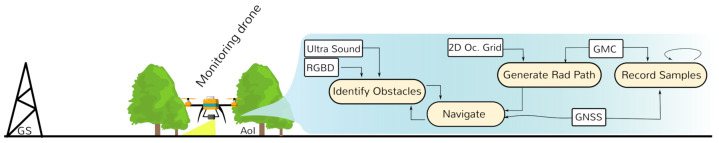
Monitoring phase—flight.

The flight operation encompasses multiple tasks, summarized in Figure 4. The drone trajectory planning is performed locally and autonomously—Generate Rad Path. It is processed on the drone’s onboard computer, using the 2D Occupancy Grid requested from the Groundstation. The drone generates a flight path presented in [29], which is optimized to:

1. cover the maximum area given the free space between obstacles,
2. get the most useful data, i.e., follow routes with high levels of radiation, and
3. achieve maximum battery range.

For navigation, the drone uses (1) the localization achieved via sensor fusion of the onboard GNSS and Inertial Measurement Unit (IMU), and (2) the target trajectory that is continuously re-computed in-flight by the onboard computer given the data acquired by the radiological sensors.

Furthermore, radiological sensors such as the GMCs need to be close to its sources to be able to detect relevant data. This sensor, performance and limitations have been described in the previous paper of the project [30]. According to our previous work, the sensors need to be at a maximum range of 1m from the ground, the drone should fly at a speed of ≈0.2 m/s and height of 1 m. The sensor is very sensitive to changes in distance from potential sources. This makes it vital to consider a height-control module that uses light-weight sensors such as ultrasound to keep the ground height as close as possible to the one defined by the operator. Furthermore this control is also important to avoid obstacles since the Occupancy Grid is height-based. Changing the height from the ground, leads to a different navigational map.

On the other hand, unexpected situations such as small tree branches not detected by the LiDAR, or GNSS errors leading to positional deviations from the intended course can lead to crashes. An obstacle identification and avoidance module is necessary to overturn the navigational module. For this, we envision the use of lightweight sensors such as the Ultra Sound and RGBD cameras. Note that in this work, autonomous navigation and obstacle navigation is not yet implemented. All drones are tele-operated, during experiments.

In parallel and during the whole flight, telemetry and radiological data are recorded for posterior processing. When the AoI is completely surveyed or the maximum range given the battery capacity is reached, the drone returns to land at its starting point.

#### 3.2.2. Post-Flight Processing

**Figure 5:**
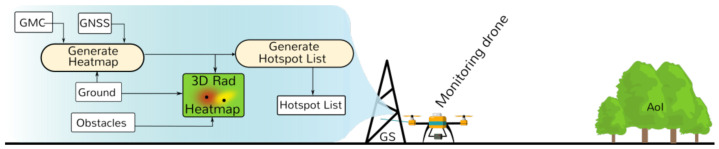
Monitoring phase—post-processing: from GMC samples to Heatmap and Hotspot list.

The process after the monitoring drone flight is summarized in Figure 5. Recorded data are downloaded to the GS. Offline, the collected GMC and GNSS data are processed. Samples are georeferenced into a local referential to match the produced 3D map. Then the heatmap is generated: the first step is to estimate position and radiological intensity of the sources and later estimate the GMC measurements in the AoI at a given height from the ground and the estimated sources. More details are presented in Section 5.4.

A 3D radiological heatmap is then composed using two elements: (1) the obstacles of the scenario (tree, rocks, etc.) and (2) the ground, colored based on the GMC heatmap. In this work, we assume radiation comes from point sources from the ground.

From the estimated sources, all local maxima are identified and the shortest route that visits each hotspot once is generated—Hotspot List (ordered). This process is known as the traveling salesman problem [31]. This route is used to perform the next stage of the mission: Inspection.

### 3.3. Inspection Phase

The third and last phase of the mission is to visit all identified hotspots, analyze the gamma-ray emission, and identify the potential radionuclides in the sources of radiation.

#### 3.3.1. Inspection Flight

The Inspection drone is equipped with a gamma spectrometer pointing downwards, and has navigation related sensors such as the GNSS, Ultrasound and RGBD camera (cf. Figure 1). The drone lands at each hotspot along the route and uses its gamma spectrometer such as a CZT to identify the radionuclides present at the location.

**Figure 6:**
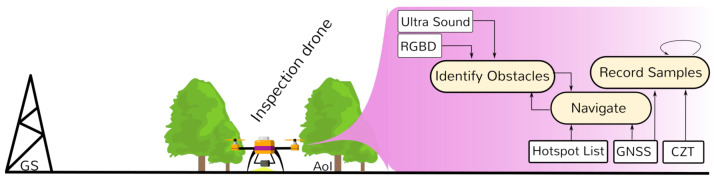
Inspection phase—flight.

The workflow of this mission stage is illustrated in Figure 6. First, given the obstacles detected in the Scouting phase, the minimum flight altitude is determined such that the drone can safely fly over any obstacle. The drone then initiates its circuit. The desired route is optimized to minimize the flight time that takes to visit all hotspots once, constrained by the possible need for battery swapping/recharging. If we predict that not all hotspots can be visited, then the hotspot list is shortened and the lowest radiation hotspots removed until the condition holds.

First, it takes-off and ascends to the minimum flight altitude. Then, moves towards the first hotspot, and lands. Upon landing, it starts measuring and recording CZT readings from the ground for as long as the operator decides for. In our previous work [30], we found that 30 min provides enough time to retrieve the signature of the radionuclides present on the ground.

After the collection terminates, it heads to the next hotspot, and the process repeats. After all points are sampled, the drone returns to the GS. In this stage of the mission, the drone should move autonomously. Note that in this work, autonomous flight was not yet implemented, and all results presented are from tele-operated flights. The major difference from this phase to the Monitoring flight phase is that instead of a path to follow, a discrete list of points is given.

#### 3.3.2. Post-Flight Processing

**Figure 7:**
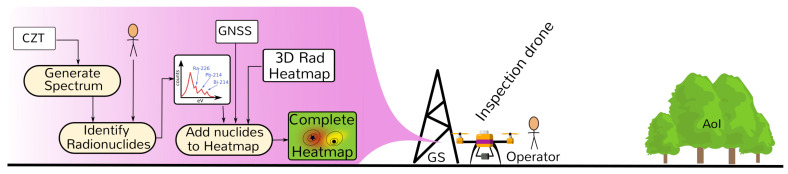
Inspection phase—post-flight processing: from CZT samples to radionuclide identification.

After the Inspection drone flight terminates, multiple procedures run on the ground station as summarized in Figure 7, starting by downloading all CZT data to the ground station. The radionuclides identification based on spectroscopy data can be done and refined given the better CPU capabilities of the ground station computer, and the results are integrated in the 3D representation with the heatmap and hotspot locations.

As shown in Figure 3, first data samples are downloaded from the drone, and, running specialized software (such as WinSpec [32]), the spectrum of each one of the measured hotspots is generated. The particular analysis of these data is not within the scope of this article. Nevertheless, at the moment radionuclide identification is done manually by qualified personnel. There is human intervention, but eventually we envision this can be performed by automatic classification algorithms.

At each hotspot, radionuclide names and energies are saved into a list. At this stage, operators should be able to open the heatmap visualizer, import such list, and finally select any hotspot to see its radionuclide sources and energies.

## 4. Functional Architecture

### 4.1. Hardware Architecture

**Figure 8:**
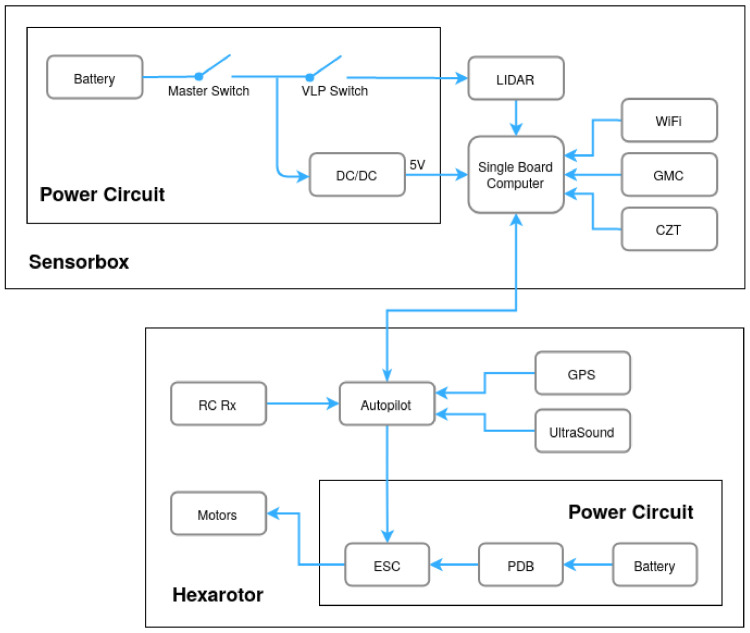
HW architecture of the Drone and Sensorbox.

All our drones are designed with two parts: (1) the vehicle itself—the multirotor—and (2) a Sensorbox, as shown in Figure 8.

The drone is powered by a battery, which is monitored and delivered by a Power Distribution Board (PDB) to the Electronic Speed Controllers (ESC) of the Motors. The target speed of each motor is dictated by the Autopilot, an off-the-shelf component closing the loop through an Inertial Measurement Unit (IMU) and GNSS. The autopilot can be used in multiple modes, including attitude control and position control. For manual control, a Remote Control receiver is connected to the Autopilot. For autonomous control, a GNSS is included for way-point navigation and an ultrasound for height measurement and control.

The Sensorbox has a single board computer in charge of collecting sensor data from multiple sensors connected to it, namely the LiDAR, GMC and CZT. Its computer can also process data during flight if necessary. Depending on the mission phase, one of the three—LiDAR, GMC and CZT—is installed. There is also a WiFi module for high-level communication with the ground station. LiDAR systems and single board computers typically operate on different voltage levels, therefore one or more DC/DC converters are normally added. The Sensorbox has its own battery due to typical LiDAR energy consumption (Velodyne VLP16 consumes 8W), but also to maintain the systems up and running, even unplugged from the drone. This way, the Sensorbox can be used independently of the drone. Besides the master power switch, there is an individual switch for the LiDAR to save energy when the device is not in use.

The Sensorbox is able to issue motion commands to the drone via a data-connection between the Computer and the Autopilot. By design, the manual Remote Control commands should always have higher priority than the Sensorbox commands. The ground station is able to interact with the Sensorbox via wireless communication, in particular using a USB WiFi dongle (IEEE 802.11).

### 4.2. Software Architecture

**Figure 9:**
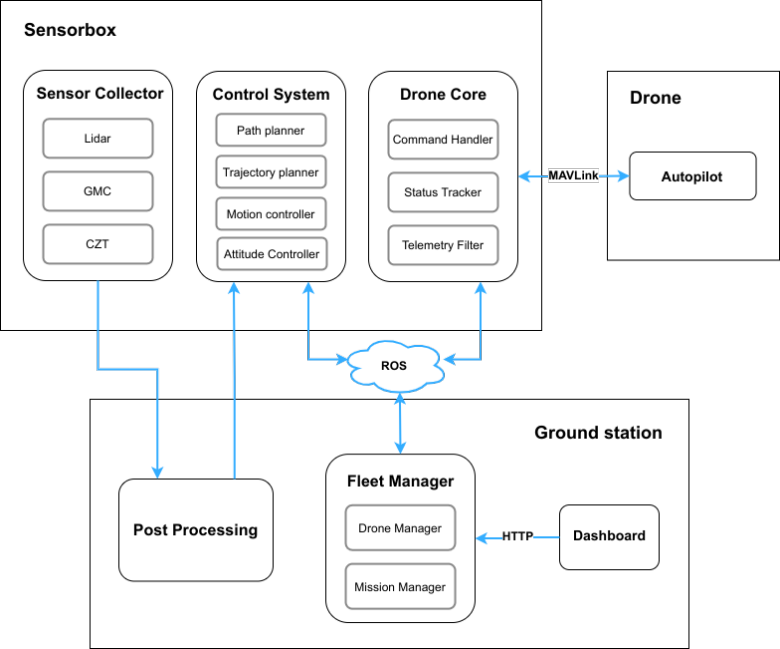
SW architecture of the Groundstation, Sensorbox and Drone.

The software architecture is supported by components that are distributed throughout the three main elements: (i) the ground station, (ii) the drone, and (iii) the Sensorbox, as shown in Figure 9. Integration with the drone is achieved through the MAVLink [33] protocol. With this protocol, we can exchange messages with the autopilot that, among other purposes, allows to receive telemetry updates and execute commands. The single-board computer is accessed through the ground station computer via command line, which allows to launch the Sensorbox software and retrieve recorded data.

#### 4.2.1. Sensorbox Software

The Sensorbox software modules run in the single-board computer and provide means for the GS components to interact with the sensors and flight controller. The Drone Core module is responsible for bridging the communication with the autopilot and external modules. It allows the configuration of some parameters, such as the drone ID, the subscribed ROS topics, the rate at which the telemetry data are published, and values like the default maximum speed or default takeoff altitude. The telemetry data are fetched at a configurable rate and published to a ROS topic by the Telemetry Filter. Subscribers such as the Fleet Manager and the Control System can retrieve the data from that topic.

There is a wide range of parameters that can be accessed through this API. Therefore, the module does not publish all of the available data, but instead it filters the data to contain the most relevant parameters. Among other data, it outputs the battery level, the flight mode, GNSS position, heading, and speed. The Command Handler subscribes a topic where the other modules can publish commands, such as arming/disarming the drone, taking off, landing, and moving to a specified location. Upon reception, these are validated by confirming if all the required parameters were provided and that the necessary pre-conditions are met, such as the drone being armed before the takeoff. After this validation, the command is sent to the autopilot to be performed.

Finally, the Status Tracker will notify the other modules of relevant events. These concern two distinct types of events: those related to tracking a command’s status or detecting other system-related events. In the case of command status, these might be used to inform when a command starts, finishes, or fails. Other events that are shared are, for example, the detection that the drone entered in manual mode, and a change in the flight controller connection status.

#### 4.2.2. Groundstation Software

The ground station software modules are launched locally in a computer and allow the user to interact with the system and process the retrieved data. The Fleet Manager module is a component of the ground station software that allows monitoring the drone state and controlling the fleet remotely. The latest telemetry data are kept at the Drone Manager, as well other relevant information, like the current command that is being executed, and if there are any errors or warnings for the drone. The user can consult this data at any time by sending an HTTP request to retrieve the data of one or multiple drones. Commands sent in an HTTP request to the Fleet Manager will be forwarded to the corresponding drone.

Missions can be submitted to the Mission Manager, which will execute the given actions accordingly. These missions will include the several actions that the drone has to perform, which may result in different execution paths at runtime—the drone may follow different paths in response to events such as a sensor reading. These may have to cross the Control System to be refined before providing clear and safe instructions to the drone, for example, considering the obstacles in the drone path.

The Dashboard is a GUI running in a web browser that communicates with the Fleet Manager in order to provide a higher-level interface for the user-drone interaction. It is possible to monitor the drone’s telemetry in the dashboard, as well as drone navigation, which is also displayed in a map. Through the dashboard, the user can send commands to a drone or submit a mission in a intuitive platform.

At a low level, the GS is able to communicate with the Sensorbox via ssh to issue commands such as initiate or close new processes and services, see disk and memory usage, list files, etc. All saved sensor data are eventually downloaded via scp, processed offline and shown in a computer on the ground, at the end of each of the Scouting, Monitoring and Inspection phases following the conceptualization presented in Section 3. The next section goes into detail regarding such algorithms.

## 5. Data Processing

In this section, we go in detail on the implementation of the main algorithms used for offline data processing, previously introduced in Section 3, namely:

1. Georeferencing—given a 3D LiDAR Odometry and Mapping (LOAM) map, the drone path (in the LOAM referential), and the drone GNSS path, we output a transformation function. This function called Georeferencing function allows any GNSS coordinate to be matched to a location in the LOAM map, and vice-versa.
2. Ground-obstacle segmentation—given a 3D map, composed of a point cloud of a given scenario, we output a classification of Ground and Non-ground for points. The first represents the floor surface, the lowest surface on the map. The second represents the obstacles above it, such as trees and rocks.
3. 2D Occupancy-grid—given a set of registered LIDAR frames, and a constant height, we output a 2D bitmap that represents the navigable area for the drone in the map at that height from the ground surface.
4. 3D Radiological Heatmap—given the 3D map, the Georeferencing function, and GMC + GNSS counts, we produce a colored 3D map of the ground based on the estimated radiation measured at that location by a person.
5. Hotspot detection—given the 3D Radiological Heatmap, we output the ordered list of the most interesting locations to inspect with CZT sensors.

### 5.1. 3D Map and Georeferencing

During the Scouting phase, drones collect thousands of LiDAR frames. As that data are downloaded into a computer, we start registering those LiDAR frames into a global coordinate system (L) using a SLAM algorithm. In this work, we used ALOAM [34], an optimized implementation of the LOAM algorithm [35]. The set of the registered frames is also referred as the 3D map of the scenario.

This 3D representation has to be linked to a system of known geographic coordinates. The coordinate system we used is the World Geodetic System WGS-84 [36], the same used by the GNSS system. There are three main reasons for such procedure. The first is that the resulting map can be compared with other 2D/3D maps using the same coordinate system. The second is that this allows other georeferenced radiological data to be located into the 3D map. Lastly, technical experts with handheld GNSS receivers can locate the hotspots represented in the map.

The LOAM algorithm returns the whole 3D representation, but also the estimated trajectory described by the LiDAR installed on the Sensorbox, and hence, the trajectory described by the UAV in the LOAM coordinate system. Given that GNSS data are also collected during the flight, we can georeference the 3D map. Let us name the LOAM coordinate system as L. Despite being similar to an East-North-Up (ENU) coordinate system, it is not generally aligned with North, East and Down directions, but aligned by the first recorded LiDAR frame.

**Figure 10:**
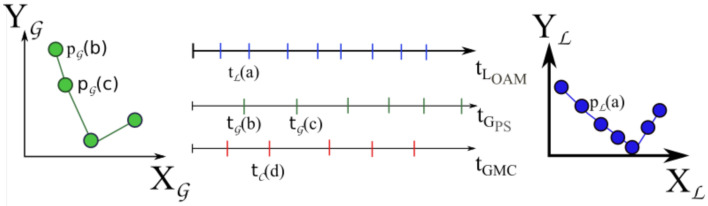
All three sensors sample at different rates. LOAM-trajectory and GNSS samples contain positions in different coordinate systems (G and L).

The LOAM trajectory sampling frequency is determined by the LOAM algorithm, and it is typically around 10 Hz. Figure 10 represents LOAM sampling events on the top, in the blue timeseries. On the right, we can see an example of the respective position values in the LOAM coordinates L. At the same time, GNSS samples are being collected, normally close to 10 Hz. Figure 10 shows this sampling in the green timeseries, in the center of the image. On the left, we can see the respective position in the GNSS coordinate system G.

Each LOAM-trajectory and GNSS message have a timestamp, which we will correlate by assuming their respective clocks are synchronized. Since the sampling frequency of the LOAM algorithm is higher than the GNSS, we start by estimating the position $p_{L}\left(a\right)$ of every LOAM message *a* in the G referential, using the following procedure:

1. Select any LOAM sample *a*: position $p_{L}\left(a\right)$, taken at time $t_{L}\left(a\right)$.
2. Find the temporal-closest GNSS sample before and after $t_{L}\left(a\right)$, namely: $t_{G}\left(b\right),t_{G}\left(c\right)$.
3. Compute $p_{G}\left(a\right)$, making a linear interpolation with respective (vector) positions ($p_{G}\left(b\right),p_{G}\left(c\right)$) using equation Equation (1).

(1) $$\begin{array}{cc} r & =\left(t_{G}\left(c\right)-t_{L}\left(a\right)\right)/\left(t_{G}\left(c\right)-t_{G}\left(b\right)\right)\in R^{+} \end{array}$$

(2) $$\begin{array}{cc} p_{L}\left(a\right) & =\left(1-r\right)p_{G}\left(b\right)+rp_{G}\left(c\right) \end{array}$$

**Figure 11:**
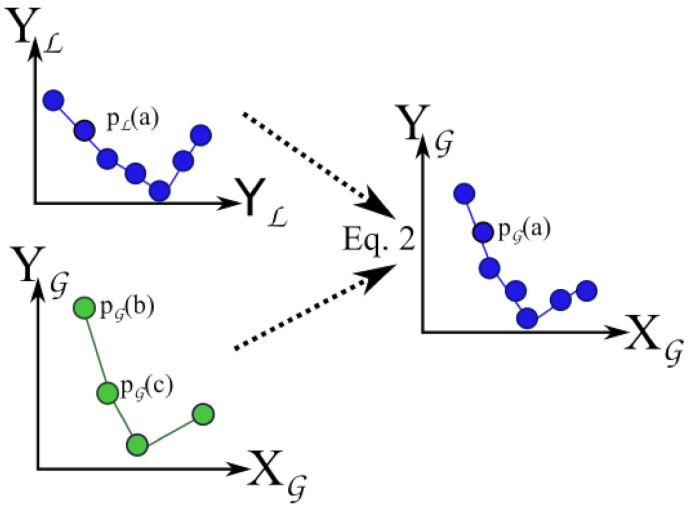
Equation (1) estimates the GNSS position of each LOAM message.

There are now two equal-sized column-vector positions $P_{G}=\left[p_{G}\left(1\right),\cdots \right]$ and $P_{L}=\left[p_{L}\left(1\right),\cdots \right]$ regarding GNSS and LiDAR data each framed in G and L coordinate systems, respectively. This process is represented in Figure 11.

**Figure 12:**
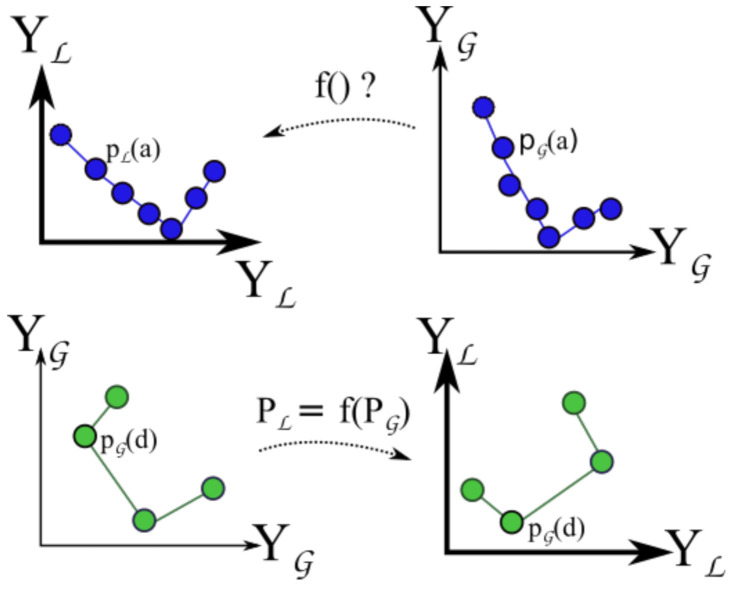
Transformation f(), from GNSS to LOAM coordinate system ($P_{L}=f\left(P_{G}\right)$).

These two vectors, also represented at the top of Figure 12, allow the conversion of a position from the GNSS (in G coordinates) to the local LOAM referential L. Such operation is represented in Figure 12 by the function $P_{L}=f\left(P_{G}\right)$. If the dataset is composed of 3 points, we find a unique solution. In general, we have numerous points and each vector subjected to different errors. Therefore, we have an over-determined system.

Function $P_{L}=f\left(P_{G}\right)$ is a composition of two functions.

First, we convert GNSS positions $P_{G}$ to a ENU referential named E-referential, yielding $P_{E}$. We use the standard function GPS2ENU (cf. Appendix A), where the centroid of the $P_{G}$ is the ENU reference point—$pr_{G}$, such that:

(3) $$\begin{array}{c} pr_{G}=\frac{1}{N}\sum_{i=1}^{N}P_{G}\left(i\right) \end{array}$$

(4) $$\begin{array}{c} P_{E}=GPS2ENU\left(P_{G},pr_{G}\right). \end{array}$$

Now that $P_{E}$ is on a similar coordinate system to $P_{L}$ (Cartesian), we can compute the translation *T* and rotation *R* that minimizes the squared error, such that:

(5) $$\min_{R,T}\left|\right|P_{L}-\left(RP_{E}+T\right)\left|\right|.$$

For this problem, we apply the method in [37], implemented in C++ by the method estimateRigidTransformation() in the PCL class TransformationEstimationSVD.

Variables *R*, *T* and $pr_{G}$ are now known, therefore function f() is defined:

(6) $$P_{L}=f\left(P_{G}\right)=R.GPS2ENU\left(P_{G},pr_{G}\right)+T.$$

At this point, GMC samples collected at the Monitoring phase can be placed into the LOAM map (cf. bottom of Figure 12). The sampling rate of GMC is 0.5 Hz, while the GNSS is 1 Hz. The first step is to estimate the GNSS location $p_{G}\left(d\right)$ for each GMC sample *d* taken at time $t_{C}\left(d\right)$ using a linear interpolation as before.

(7) $$\begin{array}{cc} r & =\left(t_{G}\left(c\right)-t_{C}\left(d\right)\right)/\left(t_{G}\left(c\right)-t_{G}\left(b\right)\right)\in R^{+} \end{array}$$

(8) $$\begin{array}{cc} p_{G}\left(d\right) & =\left(1-r\right)p_{G}\left(b\right)+rp_{G}\left(c\right). \end{array}$$

Having now a vector of GMC samples georeferenced in G, the next step is to apply f() to obtain the final transformation into L.

### 5.2. Ground-Obstacle Segmentation

For the scope of this paper, the sources of radiation are mainly assumed to be on/under ground. For radiological reasons, the UAV has to move close and ideally at low height from the ground to detect such sources, given that its radiation decays rapidly with distance. A crucial step in our work is to segment the 3D map into ground and non-ground. The segmentation allows the visualization tool to color the ground surface as a function of estimated radiological intensity on every location, while leaving above-ground-objects (such as trees) colored under a different set of rules.

Another product of segmentation is the creation of a matrix where each cell represents a squared location x,y, and its value contains the ground level *z*. This constitutes the Digital Elevation Model (DEM) of our AoI.

The set of points classified as ground should represent the surface from where objects emerge such as trees, rocks or buildings. The non-ground point cloud may encompass different things; in areas with natural sources of radiation, it is most common to find low-height trees or bushes/shrubs. Small buildings may also be present if inspection is in urban or semi-urban scenarios. However, other elements such as medium and large size rocks (e.g., in old mines) or any type of element created by the human (e.g., cars, roads, street-lamps, electricity pylons, and any type of constructions) also may appear.

**Figure 13:**
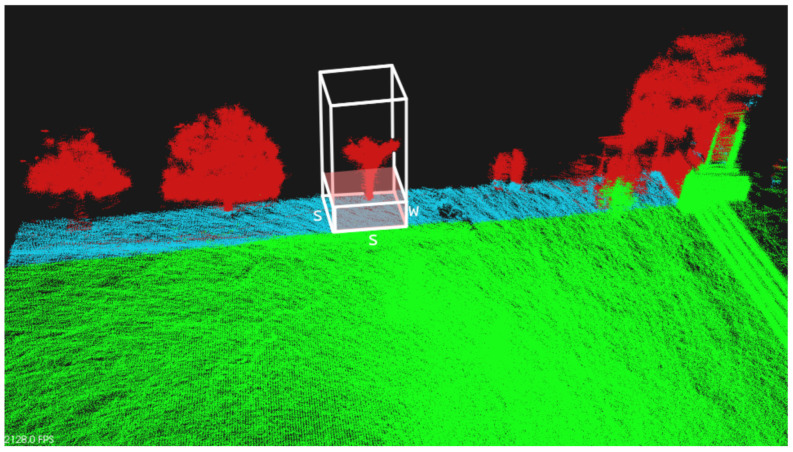
Segmentation Process. Green: points to be segmented—M. White-box: sector under analysis—S. Pink-box: candidate ground points—W. Blue: points already segmented as ground—G. Red: points already classified as non-ground.

The segmentation algorithm operates the following way. First, we identify which AoI is to be segmented within the whole generated 3D map. AoI points represent the set M. The area to be segmented is depicted in green in Figure 13. Then, one by one, the algorithm selects a small sector—a vertical squared prism with base dimensions s×s, and infinite height. In Figure 13, this sector is presented by white edges. This sector is centered at some given $x_{0},y_{0}$ coordinates, and the algorithm filters all AoI points (x,y,z) that follow the inequalities $|x-x_{0}|<s/2$ and $|y-y_{0}|<s/2$. This set of points is named S⊂M.

After this step and within S, we find the point with minimum *Z* coordinate $z_{m}$ (the lowest point), and consider a region with height *w*, such that only points (x,y,z)∈S that follow the inequality $z-z_{m}<w$ are selected. This set of points is named W and is inside the pink box in Figure 13.

For the last step, we run a RANSAC [38] algorithm on W⊂S to find the biggest plane in this region with a maximum angle of 15deg around the Z axis. The plane typically has slope. RANSAC returns its perpendicular vector *v*, and its origin (elevation) *z* (cf. vector in Figure 13). The algorithm ends up classifying some points as Ground (G⊂W), in particular all points 10 cm around such plane. All other points—S∖G—are classified as Non-ground.

When the region under analysis has a tree or other obstacles, few points are effectively detected at the ground level; With the *w*-height filter, we minimize the chance that RANSAC mistakenly find planes on tree trunks, for example.

After all x,y prismatic sectors are processed, all Ground points $G_{x,y}$ are concatenated into a single set and saved into a Ground.PCD file; all other points are classified as Obstacles and saved into Obstacles.PCD file. Furthermore, a Digital Elevation Model (DEM) file is generated for later usage by other algorithms.

Given the computational effort of this operation, it was common to run out of memory while loading the whole 3D point cloud at the same time as operations were being performed. For this reason, divide-and-conquer mechanisms were used, namely saving the map into sectors on different files, and load one by one as needed. For reference, a sector with 1 m${}^{2}$ (s=1) takes one second to process on a Lenovo Thinkpad Intel Core i5-7200U CPU @ 2.50 GHz with 8 GB of RAM.

### 5.3. Fixed-Height 2D Occupancy-Grid

As mentioned in the previous section, there is the need to compute a path for the monitoring drone to fully cover the scenario. Due to the limited sensitivity of the GMC sensor, we aim at flying at a low and constant height (1–2 m) from the ground. At this height, we typically find multiple obstacles such as trees, boulders, and electric posts that we aim to avoid during flight. In general, the number and location of obstacles depends on the height from the ground.

We intend to use the collected LiDAR data during the monitoring phase to classify the space into occupied, free and unknown areas—an occupancy 3D grid. Then, using the estimated location of the ground surface (cf. Digital Elevation Model (DEM) generated at the segmentation phase), and the desired height of flight, we generate a 2D occupancy grid. This is a subset of the 3D occupancy grid. The 2D occupancy grid is a flat map where the navigable area is determined. We finally generate a path inside this area. This path avoids obstacles within some safe margin. We assume the drone has a circular area of sensing, and the generated path must cover the whole AoI.

The first step of the algorithm is to compute 3D areas that are free. We use the Octomap [39] algorithm. This algorithm allows the user to add a LiDAR frame and its source location. The LiDAR is based on the principle of the Time-of-Flight (ToF) of a laser beam touching and echoing from the closest element in the scenario in relation to the sensor. This means, from the LiDAR sensor to the closest element in the scenario, there is no other element in between. Mathematically speaking, the conical shape that starts in the center of the LiDAR sensor and ends up on the circle painted by the laser beam on the element, represents free area. Until here, the 3D representation, with or without segmentation, does not include the free area representation, where the UAV can move though without crashing. For Guidance, Navigation and Control (GNC), this map has to be improved to include the free area, in particular for guidance, where the path is planned.

Taking into account the principle of working of LiDAR sensors, we use Octomap to classify the 3D space with three types information: (i) free area, as previously described, (ii) occupied space, where a laser reached a surface of an element, and (iii) unknown space, which corresponds to all the other space, from where there is no information, i.e., could be free or occupied). Given the worst case approach, the unknown space ends up considered as occupied area by the path planner algorithm, but provides important information to improve the scouting operation, i.e., to where the UAV should fly to expand the 3D representation of the scenario.

After this classification and given the Digital Elevation Model (DEM) generated at the Segmentation algorithm, the cubic map from Octomap is sliced, and only the cubes at a desired height from the ground are selected within some margin. We chose to use twice the height of our drones for that margin.

### 5.4. Radiological 3D Heatmap

One of our goals is to estimate the scalar field of radiation across the AoI, given a limited number *I* of locations where our GMC sensor collected radiation samples. At each one of those locations $X_{i}$, we measure its radiation value $\sigma_{i}$. Let’s consider our measurement vector Σ the set of measured radiation $\left\{\sigma_{1},\sigma_{2},\cdots ,\sigma_{I}\right\}$ at locations $\left\{x_{1},x_{2},\cdots ,x_{I}\right\}$, respectively. We assume these measurements equate to the aggregated effect of all sources of radiation present on the ground within our AoI.

A naive approach to estimate the field in any location is to interpolate the measured values in the neighborhood. In that case, we could interpolate using a weighted average giving more weight to close samples and less to farther samples. However, this would limit our capacity of estimation. If all samples are collected at a certain height from the ground, we could only properly estimate close to that same plane or surface. We assume that radiation comes from the ground, and since radiation decays with distance (inversely squared), we can predict that the radiation field at 1 cm from the ground needs to be higher than at 1 m, for instance. Furthermore, radiation is addictive: some locations can measure high radiation due to two sources nearby, or in alternative due to one strong-source nearby.

To improve estimation capacity, we model unknown sources on the ground. They can be modeled as a set of *J* locations $p_{j}$ whose radiation value $\gamma_{j}$ is unknown. Therefore, let us consider that the to-be-estimated radiation vector Γ is the set of radiations $\left\{\gamma_{1},\gamma_{2},\cdots ,\gamma_{J}\right\}$. To model the impact of each source *j* on each one of the measurements *i*, we consider that they are linear independent and their impact falls quadratically with distance (cf. Equation (9)). $d_{i,j}$ is the euclidean distance between the location of measurement *j* and a potential source *i*. $\delta_{i,j}$ is by definition equal to $d_{j,i}^{-2}$.

(9) $$\sigma_{i}=\sum_{j=1}^{J}\left(\frac{1}{d_{j,i}^{2}}\right)\gamma_{j}=\sum_{j=1}^{J}\delta_{i,j}\gamma_{j}$$

We can now describe the problem in a matrix form using Equation (10), where Δ is a matrix whose elements are $\delta_{i,j}$.

(10) $$\Sigma =\Delta \Gamma \Leftrightarrow \left[\begin{array}{c} \sigma_{1}\\ \cdots \\ \sigma_{I} \end{array}\right]=\left[\begin{array}{ccc} \delta_{1,1} & \cdots & \delta_{1,J}\\ \; & \cdots & \\ \delta_{I,1} & \cdots & \delta_{I,J} \end{array}\right]\left[\begin{array}{c} \gamma_{1}\\ \cdots \\ \gamma_{J} \end{array}\right].$$

Before starting to estimate the sources intensity, its number and locations is also unknown. Our initial approach was to assume that all ground points are potential sources. Then, for computational efficiency, we considered only ground points within a 2 m-radius sphere around GMC sampled locations. Under this assumption, we assume that GMC measurements are not affected by any possible source at more than 2 m away. This value may depend on the sensitivity of the sensor in use. To improve the computation speed, we sub-sampled all the previous points into a 3D grid with 10 cm of side (known as Voxel Filter). This should provide a good representation of the sources underground, and it is considerably more computational efficient.

To compute sources intensity, we solve Equation (10). Since Δ is in general not squared, it has no inverse. By design J>I, commonly Δ has full rank *I* and the equation system is typically under-determined (*J* unknowns, *I* linear-independent equations). Therefore, to solve such system for our unknown Γ, we try the traditional approach with the pseudo-inverse of Δ, matrix $\Delta^{†}$, as shown in Equation (11). (Pseudo-inverse can be computed using the SVD decomposition, such that if Δ = UΛV’, then $\Delta^{†}$ = V$\Lambda^{†}$U’. $\Lambda^{†}$ is the pseudo-inverse of Λ, which is formed by replacing every non-zero diagonal entry by its reciprocal, and transposing the resulting matrix.).

(11) $$\begin{array}{cc} \Gamma & =\Delta^{†}\Sigma +\left(I-\Delta^{†}\Delta \right)y\;,\;\mathrm{where}\;y\in R^{J}\;\mathrm{is}\;\mathrm{arbitrary} \end{array}$$

The pseudoinverse approach provides solutions with Minimum Squared Error. MSE=||ΔΓ−Σ|| is equal to zero in a under-determined scenario (Ignoring Equation (11)’ second term, we get the solution with the lowest norm ||Γ||.). Its major issue is that $\Delta^{†}\Sigma$ often provides negative solutions to $\gamma_{j}$, which is not physically possible in our problem. We seek *y* such that the minimum value in Γ is zero. Defining $A=\Delta^{†}\Sigma$ and $B=(I-\Delta^{†}\Delta )$, we can rewrite Equation (11) such that:

(12) Γ=A+By

Let us define column vector $C=[c_{1},\cdots ,c_{J}]$ as a function of $A=[a_{1},\cdots ,a_{J}]$, such that:

(13) $$\begin{array}{ccc} c_{i} & =0 & \;\mathrm{if}\;a_{i}>0 \end{array}$$

(14) $$\begin{array}{ccc} c_{i} & =-a_{i} & \;\mathrm{if}\;a_{i}<0. \end{array}$$

Solving C=By should lead to Γ=A+By≈A+C , which by definition should be positive ($\gamma_{j}>0,\forall j$). The last step is to compute the estimation for the GMC counts on the whole scenario. For that, we can provide a point $P_{o}$ and estimate its count $\sigma_{o}$. Given the Euclidean distance between point $P_{o}$ and all *j* sources ($d_{j,o}$), it yields:

(15) $$\sigma_{o}=\left[\begin{array}{c} 1/d_{1,o}^{2},\cdots ,1/d_{J,o}^{2} \end{array}\right]\left[\begin{array}{c} \gamma_{1}\\ \cdots \\ \gamma_{J} \end{array}\right].$$

Typically, for visualization, we show the heatmap for a grid of points every 10 cm, at h= 1 m above the ground level, but other values can be used.

### 5.5. Hotspot Detection

The location of maximum intensity of the radiation (scalar) field Σ is the location of hotspots, and therefore points-of-interest to be analyzed by the CZT. Given the map of estimated sources, we can easily detect which are the highest values in the set. To avoid inspection of points too close to each other, every time a point is classified as interesting, we remove all its close neighbors within a distance of 10 m. The list of highest values can be limited by the operator, whether by number or by threshold.

### 5.6. Visualization

A new visualization tool was created to allow operators to view and navigate interactively on the 3D reconstructed scenario. This tool allows overlaying radiological heatmaps and show detailed information of identified hotspots. The tool was implemented using C++ Pointcloud Library (PCL) [40]. It allows us to load, manipulate and visualize point clouds and meshes.

## 6. Experimental Setup

### 6.1. Drone Setup

The drone used for the experiments was a hexarotor, designed to support the weight of the smallest 3D LIDAR system we had available—Velodyne VLP16. Its mass is 1 kg, therefore we used a Tarot 960 hexacopter frame (960 mm span) with Quanum carbon fiber 33 cm propellers, providing 1.5 kg to 2.5 kg of payload capacity according to the vendor. For the motors, we used Tarot 5008/340 KV and Hobbywing XRotor Pro 40A Electronic Speed Controller (ESC). The autopilot is the Pixhawk 4 [41]. The autopilot has integrated accelerometer, gyroscope, magnetometer, and barometer, and has input for a GNSS receiver. The GNSS receiver chip is the u-blox Neo-M8N, which has an integrated magnetometer. The drone is powered by a 12,000 mAh 6S (22.2 V) Turnigy Graphene LiPo battery.

During preliminary experiments, we concluded that the drone has a maximum flight time of approximately 30 min when its payload includes the LiDAR, and 40 min without it.

The flights can be performed without interruption, i.e., without return to a safe place neither landing, if the drone has the required configuration and battery charge to pursuit its goal. However, if it is necessary to perform interruption between different flights, or even during the same flight, the drone must return to a safe place, where the operator or other member of the team is able to change its configuration, or just replace the discharged battery with a charged one. Then, the flight can be resumed returning the drone to the last position where the mission was interrupted. The drone is always in line of sight of the operator, who is in a safe place, as all the other staff.

### 6.2. Payload Setup

**Figure 14:**
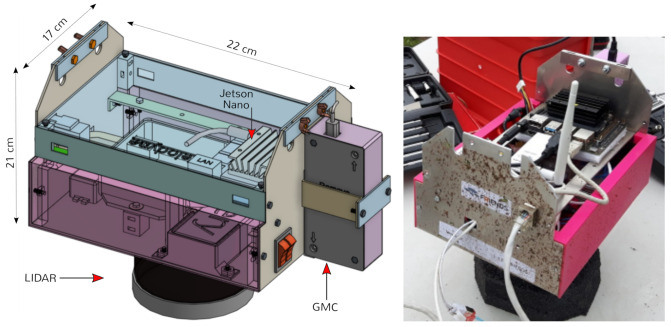
Sensorbox: CAD model (**left** image) and photo of the actual Sensorbox without lids (**right** image).

The drone payload was named Sensorbox. It is composed of its case, sensors and computer, and its electrical power system. The case was machined out of a 2 mm thick aluminum sheet, and its main purpose is to protect and carry all the hardware components. The Sensorbox can be installed under any of the drones in our fleet. All drones have two parallel bars under their chassis where the Sensorbox slides in and is secured by means of two main screws. Figure 14 shows the CAD and a photograph of the Sensorbox.

The central component is a NVIDIA Jetson Nano single-board computer [42]. The computer runs Ubuntu 18.04 and all of the required ROS Melodic software for data recording, both telemetry and sensor data. During the Scouting phase, the computer is connected to a Velodyne Puck LiDAR sensor [43] (VLP-16) through a Ethernet port. The LiDAR has a 360 horizontal field of view, with a rotation rate of 10 Hz, resulting in an angular resolution of 0.2. The official Velodyne ROS node runs to publish data, and an instance of rosbag record is issued to save data into a bag file with compression (lz4). The sensor has a vertical field of view of 30, composed of 16 channels ranging from −15 to +15 in steps of 2. With respect to the range, the authors find that results are reliable up to 50 m. The sensor generated roughly 200 MB of data every minute. A SD card with 10 GB of free space was installed in the Nano.

During the Monitoring phase, the Sensorbox is connected to the Sparkfun 11345 GMC via USB. It weights 50 g, and measures (105×44×25) mm${}^{3}$. The sensor is composed of a LND-712 [44] Geiger-Müller tube connected to a programmable microchip ATmega328p [45] microcontroller which communicates over a serial-to-USB converter. The microcontroller was configured in-house to transmit the particle counts every *T* seconds, where *T* is a configurable parameter. We chose T=2 for our missions to be the fastest sampling rate that generates no issues. The Sensorbox has also been tested with a Mazur PRM-9000 [46], a certified calibrated GMC. The PRM-9000 is considerably heavier than the Sparkfun GMC, mainly due to its built-in power source, LCD screen, case, and buzzer. Moreover, the in-house GMC has a configurable time of measurement and its window can be pointed downward without complication, increasing the sensor efficiency. Therefore, all experiments were done using the Sparkfun GMC. Again, ROS bags were used to save data.

During the Inspection phase, the Sensorbox is connected to a μSPEC 500 CZT. It is a 80 g device that measures (25×25×72) mm${}^{3}$. Its output is saved, to be later imported into specialized software to produce spectrum data. Both radiological sensors have been calibrated, and its detailed performance is described in [30].

**Figure 15:**
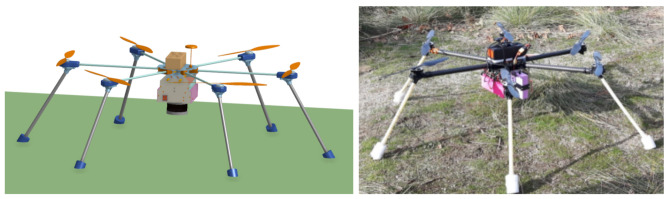
Drone and Sensorbox on the ground: CAD (**left**) and photograph, before takeoff (**right**).

All the hardware vital for drone flight is located in the drone chassis. All other systems are inside a metal case that can be easily installed and removed from the drone system—the Sensorbox. The drone is able to perform manual (Remote Control) flights without the Sensorbox, removing a single element: a USB cable between the Autopilot and the Sensorbox computer. Figure 15 depicts the concept of the drone with the Sensorbox onboard.

In addition, the Sensorbox can be easily replicated and installed with all, or only part of, the sensors in another drone. The same Sensorbox can be even used without a drone, transported by another type of vehicle, by a UGV, by an automobile or simply by humans walking. Nevertheless, the solution presented in this paper is specially designed and optimized to be installed in drones to operate in scenarios for radiological inspection.

### 6.3. Testing Scenarios

**Figure 16:**
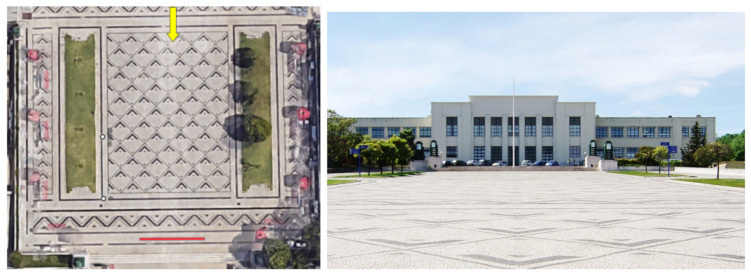
Local A: University Campus. (**Left**) Satellite view. (**Right**) Ground-level photograph.

The target scenarios are often in remote and/or abandoned places, sometimes with difficult access, where power supply and remote communication is very limited. Nevertheless, the presence of radioactive material in these places may have an impact on neighboring areas. The first scenario—Local A—tested was inside the University campus. Two controlled sources were placed inside a box, on a wide area surrounded by a dozen of trees, as shown in Figure 16. On the satellite view, a 10 m-long red marker is included for scale. A yellow arrow is added to show the position and direction of the camera to take the ground-level photograph.

**Figure 17:**
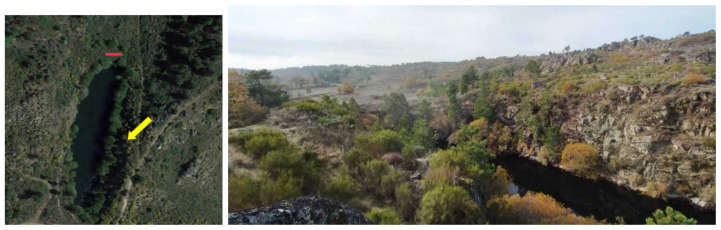
Local B: an old uranium mine. (**left**) Satellite view. (**right**) Ground-level photograph.

The second scenario—Local B—is in a old uranium mine, in the interior of Portugal. Its satellite view and a ground level photograph are shown in Figure 17. The ore extraction site is covered by water. The surroundings are hilly slopes with trees, bushes, and other vegetation. A small track is available and it is visible in the figure. The location of all the material removed in the past and considered wasteful was known, and it is represented by the red region in Figure 17.

**Figure 18:**
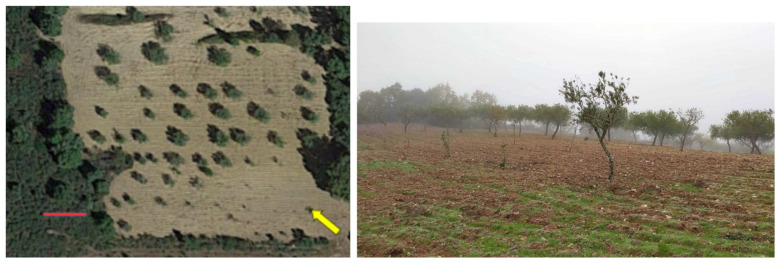
Local C: olive groove with known natural radiation. (**left**) Satellite view. (**right**) Ground-level photograph.

The third scenario—Local C—is an olive groove, with sparse trees, ranging from 50 cm to 4 m in height. Trees are separated by 3 m or more. A satellite view is shown on the left of Figure 18, and a ground-level picture is shown on the right. The ground is plowed, with only dirt and trees. The terrain is sloped but easy to walk. The map of the ground radiation was not known, but public agencies knew that there is natural radiation above background levels.

## 7. Results

### 7.1. 3D Maps

**Figure 19:**
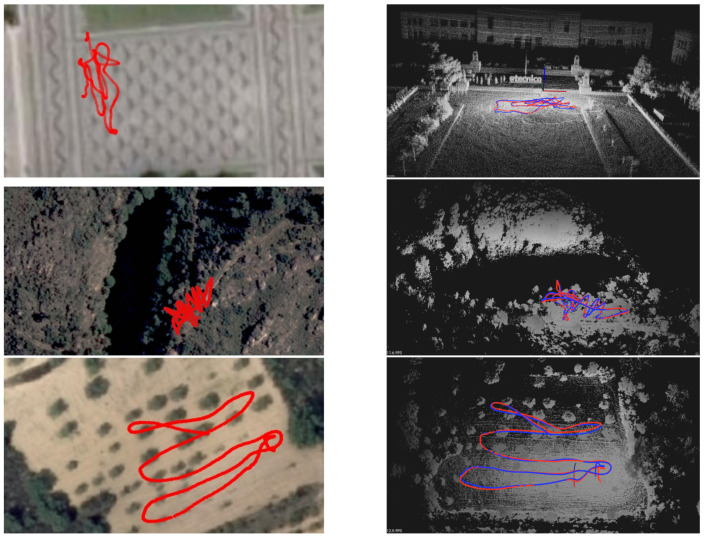
**Left**: GNSS path overlaid on satellite view. **Right**: 3D maps generated from LIDAR data running ALOAM algorithm, with lines representing estimated flight paths from LIDAR and GNSS data (blue and red, respectively). Scenarios A, B and C from top to down.

In order to obtain the 3D map, the Scouting phase was performed on each one of the three presented scenarios. The estimated traveled path for the scouting drone (equipped with LiDAR) is shown in Figure 19 in red (GNSS data) and blue (LOAM data). Each flight took between 100 s and 300 s, traveling a total (in average) of 100 and 400 m. The drone flew roughly at 10 m from the ground. Table 1 presents some details on the Scouting mission, for each flight/scenario.

Due to the high-cost of the sensor and the high-risk of its permanent damage in case of any type crash, we decided to perform tele-operated flights helped by a copilot at this stage. Nevertheless, in the future we intend to experiment using the GNSS to perform automatic flights, and the LiDAR to assess obstacles close to the drone. This way we will be able to scout farther and without line of sight, and under adversarial conditions (mist, night time, etc). We were able to reconstruct the scenarios in 3D on every run.

In Table 1, we present some of the main metrics from the LOAM data, and the time taken to pre-process such data. In general, there is roughly the same number of GNSS samples and LOAM samples. The LiDAR produces around 3–5 MB/s of data. LOAM algorithm is designed to run as fast as the LiDAR is acquiring data. However, we found that we obtain better results replaying data at a rate of 50% of the Mission time and running LOAM. The preprocessing tasks necessary before segmentation that take the most time are: outlier deletion, pointcloud transformation (rotation, translation and geo-referencing), and finally saving the pointclouds. The total time to run such tasks is similar to the duration of the scouting mission.

In local B, the lake (as expected) does not reflect the laser beam from the LiDAR, and the density of trees makes it hard to register the ground surface. Nevertheless, we where able to detect the other side of the lake moving only on the right side of it. In local A and C, the ground surface is well registered up to 40 m from the LiDAR. Moving in a snake-like pattern is not absolutely necessary as shown by the results from local A. However, due to objects such as trees, there are holes or missing data in some locations. The sweeping pattern can be improved in the future.

### 7.2. Segmentation

**Figure 20:**
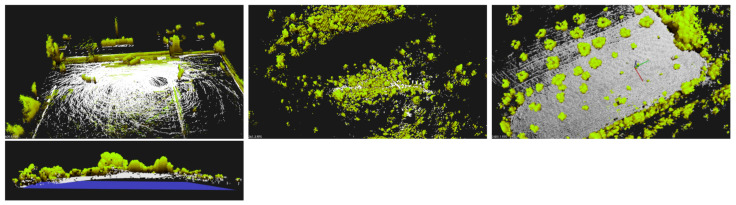
Segmented 3D maps (40 m × 40 m) into ground and non-ground data. Scenarios A (**left**), B (**center**), and C (**right**). Bottom picture shows side view from scenario C. Scale: axes are 5 m long.

After the 3D map data are pre-processed, we run a Segmentation algorithm to classify data as Ground and Non-ground. In Figure 20, we present the three maps shown in Figure 19, where the ground points are colored in white, and the non-ground data are shown in a brown-to-green palette, based on ascending height from the ground level. In general, not all ground points have the the same *Z* coordinate. The bottom image in Figure 20 shows a detail of the olive-groove scenario, where an horizontal blue plane was added to clarify the curvature of the ground surface. Nevertheless, the segmentation algorithm was able to distinguish the floor from the trees and other obstacles in all the scenarios. At the moment, segmentation is a slow process in comparison to the other processes presented in this work, taking around 1–2 s to segment a square area of 1 m^2^.

### 7.3. Octomap + 2D Occupancy Grid

**Figure 21:**
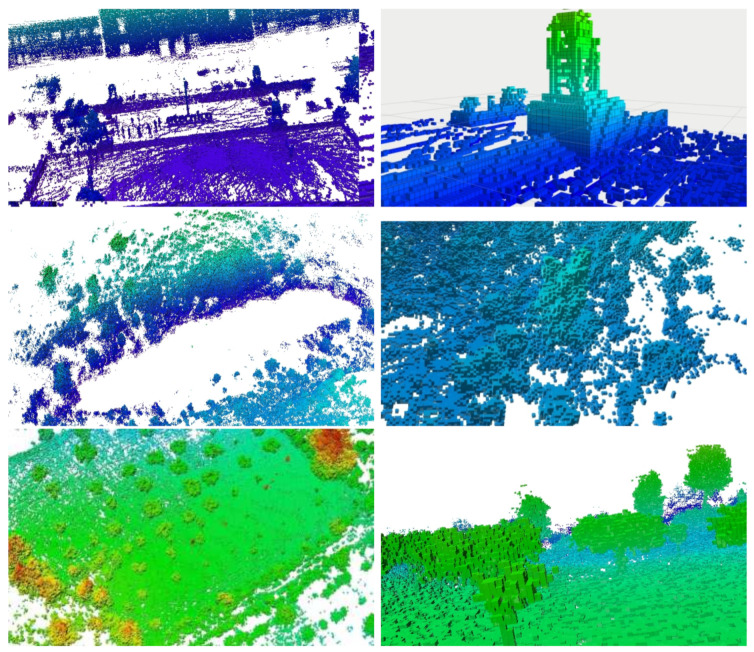
Octomaps with occupied areas from the three scenarios A (**top**), B (**middle**) and C (**bottom**). Left column presents a broad view, and the right column a detailed view.

Given the pointclouds from the three locations, we run Octomap algorithm to identify occupied, free and unknown areas for navigation goals. In Figure 21, we present the occupied areas generated by the Octomap algorithm on the three scenarios. Cubes are color-coded by its Z-coordinate (green is low, red is high). In the left column, we can see a broad, aerial view from afar. In the right column, we see the same scenarios in detail, where individual cubes are visible (side 12.5 cm).

From DEM (elevation) data, one can sweep Octomap data and confirm which cells are whether occupied/unknown or free, regarding a given height to the ground. This information is the 2D occupancy grid and it is saved into a bitmap of locations. Obstacle areas are enlarged for safety and the final map represents locations where the drone can confidently move without encountering obstacles.

**Figure 22:**
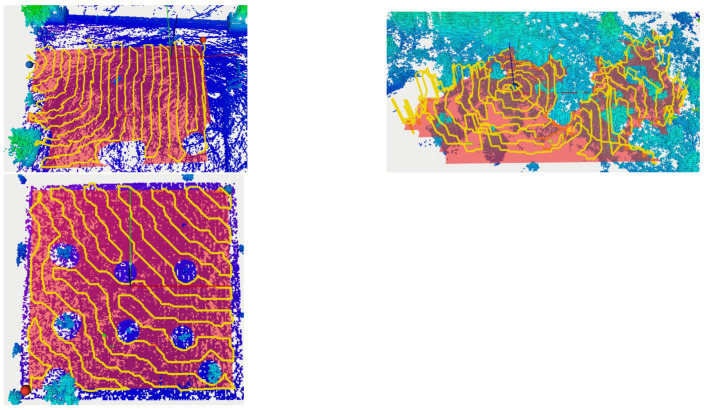
Top view of the three scenarios: represented as Octomaps (voxels in green-blue tones according to Z-axis coordinate). Yellow line represents the flight trajectory for the drone at 1.5±1 m from the ground. Red plane represents free-to-fly areas. Scenarios: Local A (**top-left**), B (**top-right**) and C (**bottom**).

This navigational map is shown as a transparent red layer in Figure 22. Note that around tree trunks, a circle is formed to represent areas where drones cannot transverse. In Figure 22, generated trajectories (based on the method [47]) are presented in yellow for the three scenarios for a height of 1.5 ± 1 m from the ground, starting at the blue sphere, and ending at the red sphere. The separation between parallel lines is set to 1 m, and it is a controllable parameter. In scenario A, it is visible that the trajectory goes around trees and covers the designed area. However, it avoids some regions without obstacles, and this is due to mission information in the Octomap structure. The LiDAR captured few points on those regions and Octomap is not able confidently return a Free state to those Voxels. In Scenario B, ground detection is harder due to tree canopies and some Octo-voxels are missing. The trajectory avoids some areas due to the unknown state of such locations. In scenario C, the computed trajectory avoids all trees and circulates around them, within a safe distance, as desired.

### 7.4. Heatmaps

**Figure 23:**
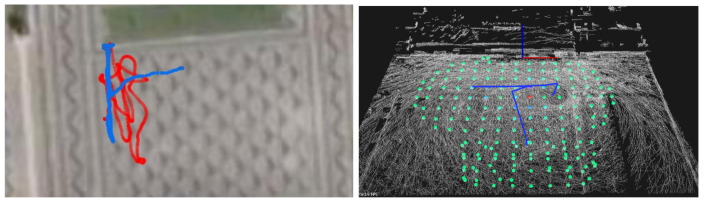
Monitoring flight in Scenario A. (**left**) Map of flight in blue, (scouting drone path in red). (**right**) 3D scenario with path and estimation of surface sources are presented by circles.

**Figure 24:**
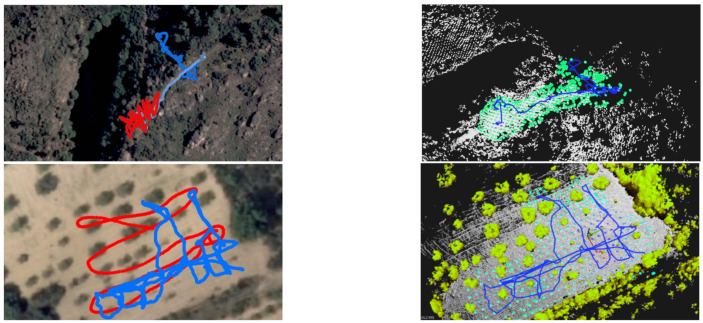
Monitoring flight in Scenario B and C. (**left**) Map of flight in blue, (scouting drone path in red). (**right**) 3D scenario with path and estimation of surface sources are presented by circles.

The monitoring drone flew roughly at 1.5 m from the ground, and collected samples every 2s with the GMC sensor. Each sample counts the number of random nuclear fissions in 2 s. Data are presented in Counts per Minute (CPM), and therefore samples are multiplied by a factor of 30. Figure 23 and Figure 24 present the flight paths as a blue line. On the left are presented 2D maps of the scenarios. On the right, one can see the 3D version of the same path, and also the candidate point-sources as small spheres on the ground surface. The red line represents the scouting drone path, as shown before. The color of each sphere represents the estimated intensity for each source Σ, ranging from low intensity (blue) to high intensity (red) (cf. method in Section 5.4). The solution for the sources intensity provided a RMSE equal to 3.12, 27.09, 34.18, to scenarios A, B and C respectively. Given the estimation of the sources Σ, one can estimate CPM measurements at any place at the ground level or any other location above it.

**Figure 25:**
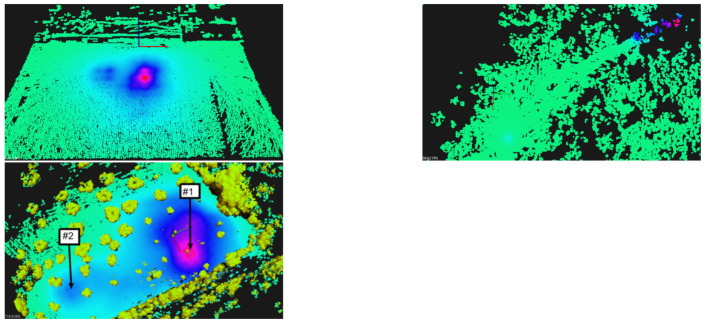
Heatmap: CPM estimation at h=1 from the ground, at each one of the scenarios. **Top-left**: Scenario A (max 109 CPM). **Top-right**: Scenario B (max 272 CPM). **Bottom**: Scenario C (max 347 CPM).

For visualization purposes, it is an improvement to show the 3D scenario with the heatmap overlaid. Figure 25, shows a 3D perspective of the floor painted in a light-blue to purple-pink gradient The color represents the CPM estimation for scenarios A, B and C when at h=1 m above the ground. Furthermore, we present obstacles (mainly trees) painted in brown-green gradient. We hide those obstacles in local A and B for clarity. In this figure, pink represents higher CPM count and light-blue shows lower GMC count.

In scenario A, one see a hotspot which matches the artificial source inside a cardboard box we have placed on the floor at that location. In scenario B, one observe a main hotspot not far from the track. It was identified as coming right under a small shrub, and it is close to the expected area of high radiation. In scenario C, one can see two hotspots. The stronger one of the right comes from under a small olive tree and we named it location #1. The one on the left is weaker and close to the edge of the olive groove, we named it #2.

**Figure 26:**
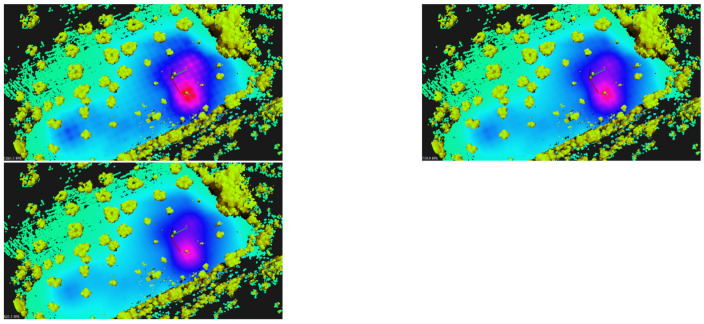
Heatmaps: GMC estimation in Counts per Minute from local C when at 1 m, 1.5 m, and 2 m above the ground. Maximum estimated value is 240 CPM, 195 CPM and 171 CPM respectively.

It is expected that increasing the height from the ground provides lower GMC counts. In Figure 26, we have the estimation in Counts per Minute from local C when at 1, 1.5 and 2 m above the ground. It is noticeable the fuzziness increases with height, as expected. Note that maximum CPM value also decreases. At h=1 m, it was measured 347 CPM; at h=2 m, one estimate an activity 25% lower, around 260 CPM.

**Figure 27:**
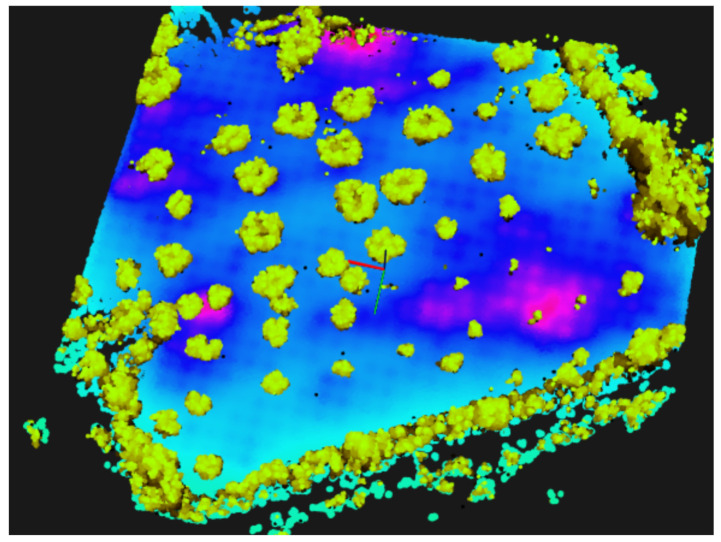
Heatmaps: GMC estimation in Counts per Minute from local C when at 1 m above the ground. Data collected on foot. Maximum estimated value is 164 CPM.

For a fair comparison, data from LiDAR and GMC was collected at the Sensorbox while being carried by a person. The Sensorbox was approximately at 1 m from the ground, and the walking speed was around 1 m/s, and the path was of the boustrophedon type. Figure 27 shows the estimated GMC field at 1m from the ground. The first visible difference is that in this experiment the range of LiDAR samples is greater than when using the drone. In this experiment, the person moved farther from the Groundstation that the drone did. There is more detail on the ground surface, and less of the top of the trees, as expected.

Regarding the heatmap, it is clear that we identified the same two hotspots as before. However, the estimated maximum CPM value is lower—164 CPM, in comparison with 240 CPM with the drone. The shape of the heatmap around the biggest hotspot is also different, and less oval. Due to greater range of data collection, one can see other hotspots at the top of the figure.

### 7.5. Hotspot Identification

For hotspot identification mission, we e previously identified the most interesting points. The selected points are all the sources with intensity above a 0.5 threshold, following the procedure explained in Section 5.5. Given the similarity of results between scenarios, we present here the results of scenario C—the olive groove. In the bottom of Figure 25, we show the selected locations for further inspection via CZT at scenario C.

The measurements were performed using a Ritec uSPEC500 CZT with a sensitive volume of 500 cm^3^. The CZT sensor was placed in the ground near the hotspot location for the three selected hotspots, with an acquisition time of approximately 32 min. The background spectrum was also measured at a location near, but outside the olive grove, aiming to compare the gamma emissions inside and outside the grove.

**Figure 28:**
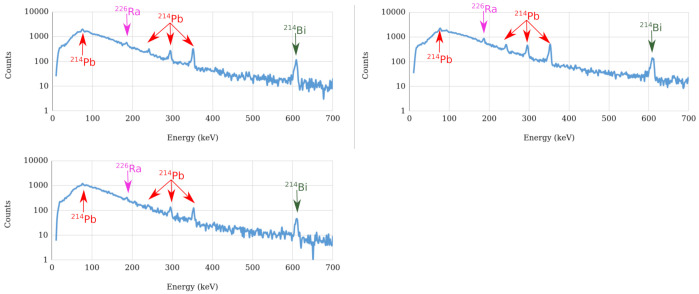
Spectra acquired with the CZT at Hotspot #1,#2 and #3, in Scenario C. The peaks identified using InterSpec [48] are marked with arrows. The acquisition time was approximately 32 min.

The data was collected using the WinSpec software provided by the CZT manufacturer and the radiation spectra acquired were analyzed with the InterSpec v1.0.6 software [48]. Figure 28 shows the spectra acquired at the three considered hotspots. Interspec was used to identify the peaks present in the spectra. For the three hotspots, seven energy peaks (X-ray and gamma emissions) from uranium-238 decay products were registered, accounting for the identification of ${}^{214}$ Bi, ${}^{214}$ Pb and ${}^{226}$ Ra.

**Figure 29:**
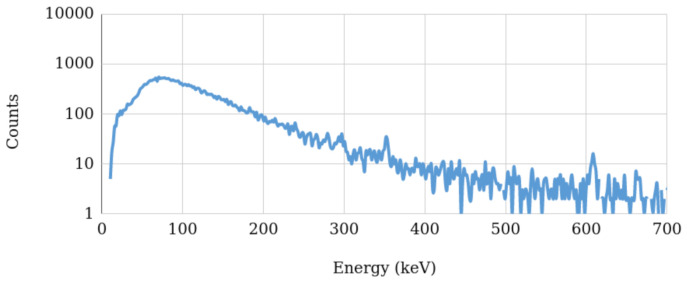
Spectrum acquired with the CZT at the background level, in Scenario C. The acquisition time was approximately 32 min.

The characteristics of the photopeaks identified can be consulted in Table 2. Hotspot #3 had the lowest particle emission rate with respect to hotspot #1 and #2, resulting in less photopeaks being identifiable in the spectrum acquired at hotspot #3. Furthermore, since the sensor detects fewer particles, the uncertainty value is higher, which can impair the proper distinction between two overlapped photopeaks. Figure 29 shows the spectrum acquired at the background level. Comparing the spectra acquired at the hotspots and at the background radiation level, we can see that the almost none of the photopeaks are identifiable in the background spectrum (only the photopeak at 609 keV can be seen), thus validating that the olive grove had an increased level of environmental radioactivity, with respect to the bordering terrains.

## 8. Conclusions and Future Work

In this paper, we presented a three-tier design to perform radiological missions with a fleet of drones. First, we have used a drone with LiDAR to scout the scenario and create interactive 3D maps. Then, we have used drones to collect GMC data samples, from which we were able to create heatmaps, overlay such data on 3D maps and identify the hotspots, the locations with the highest radiation. Finally, we were able to inspect in detail some of those locations, and compute their radiation spectra to identify the radionuclides. We conclude that drones are a viable tool to scout and monitor scenarios faster and as reliable as personnel on the ground.

In the future, we will focus on developing autonomous flights. For the scouting phase, we intend to experiment using the GNSS to perform automatic flights, and feed live LiDAR data to the drone to assess obstacles close-by. For the monitoring phase, we will focus on constant and low-height flight by integration of vertical navigation control, obstacle avoidance hardware and state-of-the-art algorithms. For the inspection phase, we will address autonomous landing at hotspots, specially when they are covered by an obstacle (e.g., a tree whose branches arch over the hotspot).

The paper is focused in radiological scenarios. However, the presented solutions and decisions are also applicable to other areas of CBRN threats. We intend to explore, in the future, other types of sensors for other CBRN scenarios, (e.g., thermal, gas such as methane, etc.) such as landfills and similar scenarios.

## Figures and Tables

**Table 1:** Scouting phase: LOAM data properties, and pre-processing performance.

Metric	Scenario A	Scenario B	Scenario C
Flight time	106 s	165 s	265 s
Bag size	296 MB	601 MB	1.3 GB
Area	180 m × 200 m	220 m × 220 m	140 m × 120 m
#Frames LiDAR	538	1532	2346
#Frames GNSS	531	1459	1110
Running LOAM	212 s	330 s	550 s
Pre-processing:	106 s	176 s	281 s
– Load Bag	15 s (7.31 M)	10 s (15.8 M)	30 s (26.22 M)
– Remove Outliers	≈29s (6.7 Mpts)	≈70 s (14.6 Mpts)	≈128.0 s (24.2 Mpts)
– Transformations	≈21 s	≈48 s	≈82 s
– Save into PCD	41 s	48 s	41 s

**Table 2:** Characteristics of the photopeaks identified in the spectra acquired from the three selected hotspots and background level.

Photopeak (keV)	Radionuclide and Decay Mode	Identifiable in Spectra from			
		Hotspot #1	Hotspot #2	Hotspot #3	Background
74.82	${}^{214}$Pb X-ray	🗸	🗸	🗸	×
92.32	${}^{214}$Bi X-ray	×	🗸	×	×
186.21	${}^{226}$Ra gamma	🗸	🗸	🗸	×
242.00	${}^{214}$Pb gamma	🗸	🗸	×	×
295.24	${}^{214}$Pb gamma	🗸	🗸	🗸	×
351.93	${}^{214}$Pb gamma	🗸	🗸	🗸	×
609.31	${}^{214}$Bi gamma	🗸	🗸	🗸	🗸

## Data Availability

Datasets are available by request. Find more info at ipfn.tecnico.ulisboa.pt/FRIENDS.

## Appendix A

To convert coordinates from geodesic (GNSS) to ENU, there are two steps. First, one converts data from geodetic (latitude ϕ,longitude λ, altitude *h*) to ECEF coordinates:

(A1) $$\begin{array}{cc} X & =\left(N(\phi )+h\right)\cos\phi \cos\lambda \end{array}$$

(A2) $$\begin{array}{cc} Y & =(N(\phi )+h)\cos\phi \sin\lambda \end{array}$$

(A3) $$\begin{array}{cc} Z & =\left(\frac{b^{2}}{a^{2}}N\left(\phi \right)+h\right)\sin\phi \end{array}$$

where

(A4) $$N\left(\phi \right)=\frac{a}{\sqrt{1-e^{2}\sin^{2}\phi }}$$

and

(A5) $$e^{2}=1-\frac{b^{2}}{a^{2}}.$$

Second, we convert data from ECEF to local ENU coordinates, using reference point $p_{R}$. $X_{r}$, $Y_{r}$, $Z_{r}$ are ECEF coordinates of the reference point. $\phi_{r}$, $\lambda_{r}$ are its latitude and longitude:

(A6) $$\left[\begin{array}{c} x\\ y\\ z \end{array}\right]=\left[\begin{array}{ccc} -\sin\lambda_{r} & \cos\lambda_{r} & 0\\ -\sin\phi_{r}\cos\lambda_{r} & -\sin\phi_{r}\sin\lambda_{r} & \cos\phi_{r}\\ \cos\phi_{r}\cos\lambda_{r} & \cos\phi_{r}\sin\lambda_{r} & \sin\phi_{r} \end{array}\right]\left[\begin{array}{c} X-X_{r}\\ Y-Y_{r}\\ Z-Z_{r} \end{array}\right].$$
